# Stable Transmission of DNA Methylation Epimutations from Germlines to the Liver and Their Association with Fatty Liver Disease in Medaka

**DOI:** 10.21203/rs.3.rs-6010210/v1

**Published:** 2025-02-14

**Authors:** Sourav Chakraborty, Santosh Anand, Xuegeng Wang, Ramji Kumar Bhandari

**Affiliations:** University of Missouri; University of Missouri; South China Normal University; University of Missouri

**Keywords:** Transgenerational inheritance, Epigenetics, Fish, Germline transmission, Environmental stressor

## Abstract

**Background:**

Environmental stressors can induce heritable traits in organisms across phyla, with distinct epigenetic alterations in gametes and phenotypic outcomes across several generations. However, the mechanisms underlying such intergenerational inheritance, mainly from the germline to the germline and from the germline to the soma, are enigmatic, given that postfertilization embryos and germline cells reprogram the epigenome in each generation to gain their cellular identity. Here, we report stable germline transmission of differential DNA methylation alterations (epimutations) and their associations with nonalcoholic fatty liver disease (NAFLD) in medaka exposed to a model estrogenic chemical but a ubiquitous environmental contaminant, bisphenol A (BPA).

**Results:**

Ancestral BPA exposure in the F0 generation led to advanced NAFLD in the unexposed grandchildren generation (F2) of medaka. The F2 liver transcriptome and histopathology revealed a severe NAFLD phenotype in females. Whole-genome bisulfite sequencing of the sperm and liver revealed a gradual shift in promoter methylation from F0 sperm (hypomethylated) to F1 sperm (mix of hypo- and hypermethylated) and F2 liver (predominantly hypermethylated). Many differentially methylated promoters (DMPs) overlapped in F0 sperm, F1 sperm, and F2 liver, regardless of sex. In females, stable transmission of 1511 DMPs was found across three generations, which are associated with protein-coding genes, miRNAs, and others and linked to NAFLD and nonalcoholic steatohepatitis (NASH). Among them, 27 canonical genes maintained consistently hypermethylated promoters across three generations, with significant downregulation of their expression and enrichment in NAFLD-related pathways, mainly fat digestion, glycerolipid metabolism, and steroid biosynthesis.

**Conclusions:**

The present results demonstrate stable inter- and transgenerational germline-to-germline and germline-to-soma transmission of environmentally induced DNA epimutations with F0 and F1 gametic epimutations, predicting the F2 liver phenotype—a clear transgenerational passage of the disease phenotype in medaka.

## Background

Diseases and health conditions can often have complex origins, including nongenetic (epigenetic) factors[1]. Epigenetic alterations, such as changes in DNA methylation patterns, can play a crucial role in how genes are expressed and regulated. The addition of a methyl group to cytosine–phosphate–guanine dinucleotides (CpGs) controls gene expression depending on the methylation pattern in the promoter region[2]. DNA methylation plays an important role in the development of disease phenotypes both *in vivo* and *in vitro*[3–6]. Vertebrate sperm maintain a high level of DNA methylation and carry molecular signatures of paternal experiences in the form of differential DNA methylation or other epigenetic alterations to offspring[7]. Paternal exposure or dietary changes can reprogram the epigenome of sperm by altering DNA methylation[8], noncoding RNA[9–11] and aberrant histone methylation and retention[12, 13]. These observations indicate that environmental factors induce epigenetic alterations in sperm, which are intricately connected to transgenerational inheritance.

Studies over the past few decades have suggested that transgenerational epigenetic inheritance (TEI) can occur in many organisms and that a wide range of environmental factors can induce TEI, including toxicants[14], stress[15], and nutrition[16]. Environmentally induced epigenetic modifications and their potential memory in the germline may influence epigenetic traits across generations[17, 18]. However, the plausible mechanism of environmentally induced epigenetic inheritance is enigmatic. Takahashi et al. provided experimental evidence that artificially introduced epigenetic changes can be transmitted across generations in mice, indicating that epigenetic alterations are inter- and transgenerationally heritable[19]. It is unclear whether transgenerationally inherited epigenetic memories are drivers of the transgenerational phenotypes that develop due to ancestral life experiences.

The period of germ cell differentiation is an epigenetically dynamic state during which external environmental stressors can establish exposure-specific epigenetic marks in the germline epigenome that can persist across several generations[20]. Among several environmental stressors, bisphenol A (BPA) is a global environmental contaminant known for its endocrine-disrupting effects and harmful properties that can be epigenetically transmitted[21]. Studies have demonstrated that BPA-induced DNA methylation patterns are associated with altered reproductive and metabolic disease phenotypes[22–25]. In mice, BPA-induced DNA methylation of lipogenic genes has been linked to hepatic steatosis[26]. Evidence suggests that direct BPA exposure can reprogram fat metabolism genes in the liver, promoting nonalcoholic fatty liver disease (NAFLD)[27]. In addition, BPA has been found to induce epigenetic modifications in sperm, leading to transgenerational reproductive disease, obesity[28], cardiotoxicity[29], glucose intolerance[30], and reduced male fertility[31]. In medaka fish, ancestral BPA exposure (10 μg/L) during the period of epigenetic reprogramming of germ cells led to NAFLD[1] and polycystic ovary syndrome (PCOS)[32] in unexposed offspring four generations later. Evidence suggests that ancestral BPA exposure can promote a transgenerational disease phenotype via germline transmission. Given that a gateway for the correction of altered epigenetic changes exists in germline and somatic cells during early embryo development[33–35], questions as to how ancestral epigenetic marks in germ cells bypass the reprogramming of the epigenome and how transgenerational passages program transcriptomes in somatic cells, leading to a disease phenotype in subsequent generations, remain unanswered. Using medaka fish as an animal model, BPA, a known environmental contaminant that induces fatty liver disease transgenerationally, and integrated epigenomic (methylome) and transcriptomic analysis approaches, we determined the stable transmission of differential DNA methylation memories (DNA epimutations) from grand paternal sperm (F0) to paternal sperm (F1) and finally to grand-offspring liver cells (F2) after embryonic exposure of the F0 ancestor. This transgenerational transmission of DNA epimutations was associated with corresponding transcriptional alterations and pathways to NAFLD in the unexposed offspring of medakas. The identified grandparental DNA epimutations were predictive of NAFLD in grand offspring, suggesting that the present results provide valuable insights into their transgenerational inheritance and their role in contributing to liver pathology in subsequent generations.

## Results

### Sex-specific NAFLD phenotype developed in grand-offspring from BPA-exposed lineage

**Figure 1:**
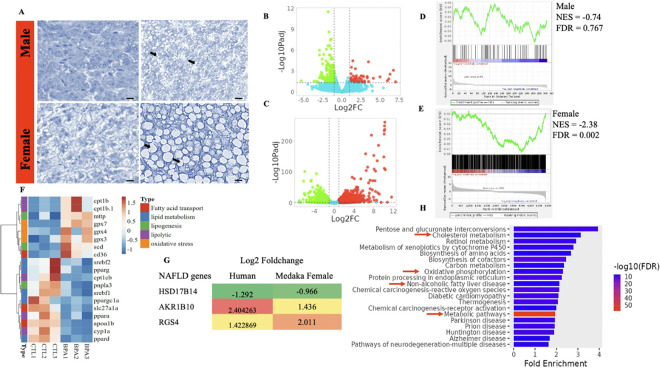
Ancestral BPA exposure induces a NAFLD-like phenotype, with more pronounced effects in female fish. **A** Histopathological examination of the liver in control lineage males and females revealed normal hepatocytes. In contrast, vacuolated hepatocytes were found in the BPA lineage, with females displaying a more severe phenotype than males do (40X magnification and scale length of 20 μm). Volcano plots showing differentially expressed genes in the livers of BPA-lineage male **B** and female **C** fish compared with those in the control lineage. **D** The horizontal dotted line corresponds to FDR = 0.05 or (−log10 Padj = 1.3). (D & E) GSEA of liver DEGs via the Harmonizome NAFLD gene set revealed negative enrichment, with females **E** showing significantly greater enrichment than males. **F** Heatmap of significantly expressed known lipolytic, lipogenic, lipid metabolism, fatty acid transport, and oxidative stress genes in the livers of females of the BPA and control (CTL) lineages. **G**Comparison of the differential expression profile (log2-fold fold change) of shared genes from the female liver RNA-seq dataset with the validated 25-gene signature for steatohepatitis and fibrosis in human patients. **H** Pathway analysis of female liver DEGs revealed significant enrichment in NAFLD-associated pathways, including cholesterol metabolism, metabolic pathways, and oxidative phosphorylation.

To assess the disease phenotype, histopathological analysis was performed on male and female livers from the BPA and control lineages of the grand offspring (F2 generation). In the BPA lineage, histological examination of the liver revealed microvesicular steatosis in males (Fig. 1A) and macrovesicular steatosis in females (Fig. 1A). The livers of the BPA lineage females developed a more severe NAFLD phenotype than that observed in BPA lineage males. Next, we analyzed the gene expression patterns associated with the observed histological phenotype. Significant gene expression changes were detected in the livers of BPA-lineage males (Fig. 1B) and females (Fig. 1C), with females showing markedly higher significance levels (FDR values) than males. Using the Harmonizome NAFLD dataset[36] and gene set enrichment analysis (GSEA)[36], we investigated whether the significantly differentially expressed genes (DEGs) in the livers of BPA-lineage males and females presented gene expression patterns correlated with those reported for human NAFLD. According to the GSEA results, DEGs in the male liver presented a relatively low degree of enrichment (NES = −0.74), with no significant correlation (FDR = 0.767) with NAFLD (Fig. 1D). In contrast, the DEGs in the livers of females were significantly enriched with NAFLD (NES = −2.38, FDR = 0.002) compared with those of controls (Fig. 1E). GSEA combined with the Harmonizome NAFLD dataset and histological findings demonstrated that the livers of BPA lineage females developed more severe NAFLD than their male counterparts. Therefore, we focused on female liver data for further analysis and characterization.

To assess potential dysregulation of fat metabolism, lipolysis, lipogenesis, fatty acid transport, and oxidative stress-related processes, we examined the expression of key genes involved in these pathways in the livers of females in the BPA lineage (Fig. 1F). All oxidative stress response genes (*gpx7, gpx4, gpx3*) were upregulated, whereas fat-metabolizing genes (*srebf2, pparg, ppard*) were downregulated. Other gene categories involved in lipolysis (*ppara, cpt1b*), lipogenesis (*mttp, scd, pnpla3*), and fatty acid transport (*apoa1b, cd36*) were also significantly dysregulated (Fig. 1F). Next, we compared our findings with published data on a 25-gene signature associated with steatohepatitis and fibrosis in human patients[37]. In the livers of BPA-lineage females, three signature genes*—HSD17B14, AKR1B10*, and *RGS4*—exhibit expression fold-change patterns similar to those observed in advanced stages of NAFLD in human patients (Fig. 1G). Furthermore, global alterations in the transcriptional profile of the livers of BPA-lineage females resulted in significant enrichment of DEGs related to NAFLD-associated pathways, including cholesterol metabolism, oxidative phosphorylation, and metabolism (Fig. 1H). Taken together, these results indicated that DEGs identified via RNA-seq were significantly linked to NAFLD pathogenesis in the livers of BPA-lineage females in the F2 generation.

### Global DNA methylation profiling of F0 sperm, F1 sperm, F2 male and female livers

**Figure 2:**
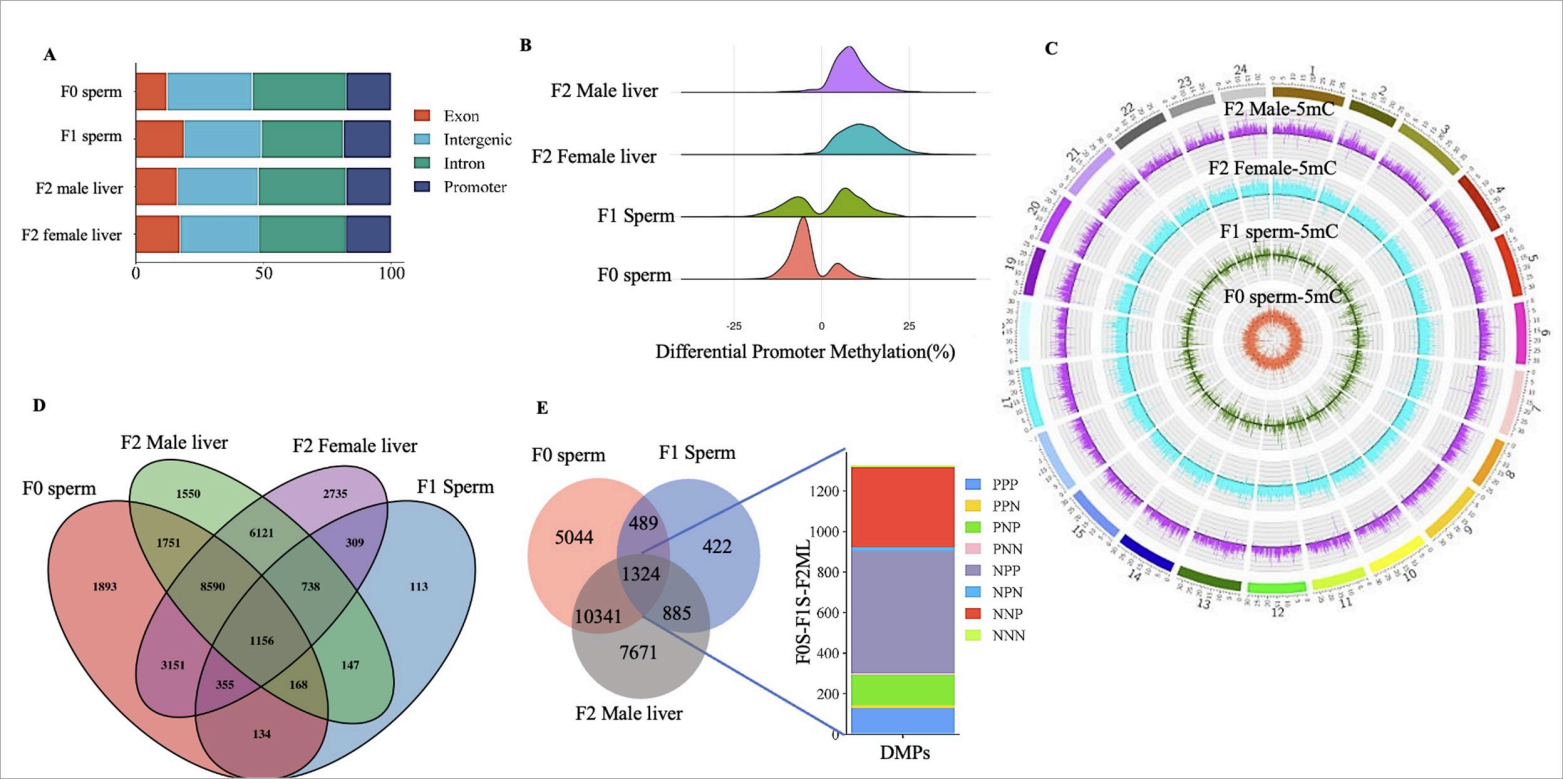
Genome-wide profile of differentially methylated regions in F0 and F1 sperm, as well as F2 male and female livers. **A** Classification of genome-wide significant differentially methylated regions (DMRs) at a 100-bp resolution. **B**Rideline plot depicting the density distribution of significantly differentially methylated promoters (DMPs). **C** Circos plot showing the chromosomal distribution of significant DMPs. **D** Venn diagram showing the overlap of DMPs across generations and sexes. **E** Venn diagram showing the overlap of DMPs among F0 sperm, F1 sperm, and F2 male livers. The common DMPs are further categorized as P (hypermethylated) or N (hypomethylated) in each individual sample. For example, an NPN category indicates that the DMP is hypomethylated (N) in F0 sperm, hypermethylated (P) in F1 sperm, and hypomethylated (N) in F2 male liver.

The global methylation profiles of the control and BPA lineages were analyzed via a 100-bp tiling window approach with a false discovery rate (FDR) of 0.1. The percentage of genome-wide methylation was significantly higher in the BPA lineage than in the respective controls for the sperm of the F1 father and the livers of F2 males and females (one-sided Wilcoxon rank sum test p value < 2.2e-16 for all; Supplementary Fig. 1A-B). Additionally, the difference in methylation percentages between the BPA lineage and their respective controls was more pronounced in the F2 female livers than in the F2 male livers (Supplementary Fig. 1B). Conversely, the sperm of the BPA lineage F0 ancestor (BF0S) presented a significantly lower percentage of methylation than did the control (p value < 2.2e-16; Supplementary Fig. 1A). Next, genome-wide differentially methylated regions (DMRs) were identified and analyzed on the basis of their genomic locations and categorized into four groups: promoter, exon, intron, and intergenic regions (Fig. 2A). Overall, the genomic location profiles of DMRs are similar except for a slight decrease in the exonic regions of F0 sperm.

### Ancestral BPA exposure-induced differential promoter methylation in F0 and F1 sperm was transmitted to the liver cells of the F2 generation.

Since promoter methylation regulates gene expression patterns[38], we analyzed the density profile of differential methylation percentages (%) across the promoter regions in F0 sperm, F1 sperm, and F2 livers of males and females via a ridgeline plot (Fig. 2B). A gradual shift in differential promoter methylation patterns was observed from F0 generation sperm to F2 generation livers. In the F0 generation, promoters were predominantly hypomethylated (negative differential methylation), whereas in the F1 generation, promoters displayed a more balanced mix of hypo- and hypermethylation. In the F2 generation, the promoters were almost exclusively hypermethylated. The differential promoter methylation pattern was uniformly distributed across various chromosomes, as shown in the Circos plot (Fig. 2C).

To track the germline transmission of the BPA-specific differentially methylated promoters (DMPs) to somatic cells of the liver of the offspring in the F2 generation, DMPs in the F0 sperm and F1 sperm were compared with those found in the livers of the BPA-lineage males and females in the F2 generation. We identified 1156 overlapping DMPs in F0 sperm, F1 sperm, and F2 male and female livers (Fig. 2D). This result suggested that some of the ancestrally established DMPs in the F0 sperm were transmitted F1 sperm via germline-to-germline transmission, which were subsequently inherited by F2 somatic cells (liver in the F2 generation of the BPA lineage) via germline-to-soma transmission, regardless of sex. In males, 1324 shared DMPs were identified in the livers of F2 males, F1 sperm, and F0 sperm (Fig. 2E). To qualitatively assess the inheritance of DMPs in the BPA-lineage liver, the DMPs in each generation were categorized as hypermethylated (P) or hypomethylated (N). Inherited DMPs that remained hypermethylated throughout F0 sperm, F1 sperm and the liver of the F2 offspring were classified as PPP, whereas those that were established in F0 sperm as hypermethylated but were subsequently hypomethylated in F1 sperm and remained hypomethylated in F2 offspring’s liver were classified as PNN (Fig. 2E). Similar annotations were assigned for other combinations, as shown in Fig. 2E.

### Inherited DMPs in the female liver are associated with NAFLD-NASH

**Figure 3:**
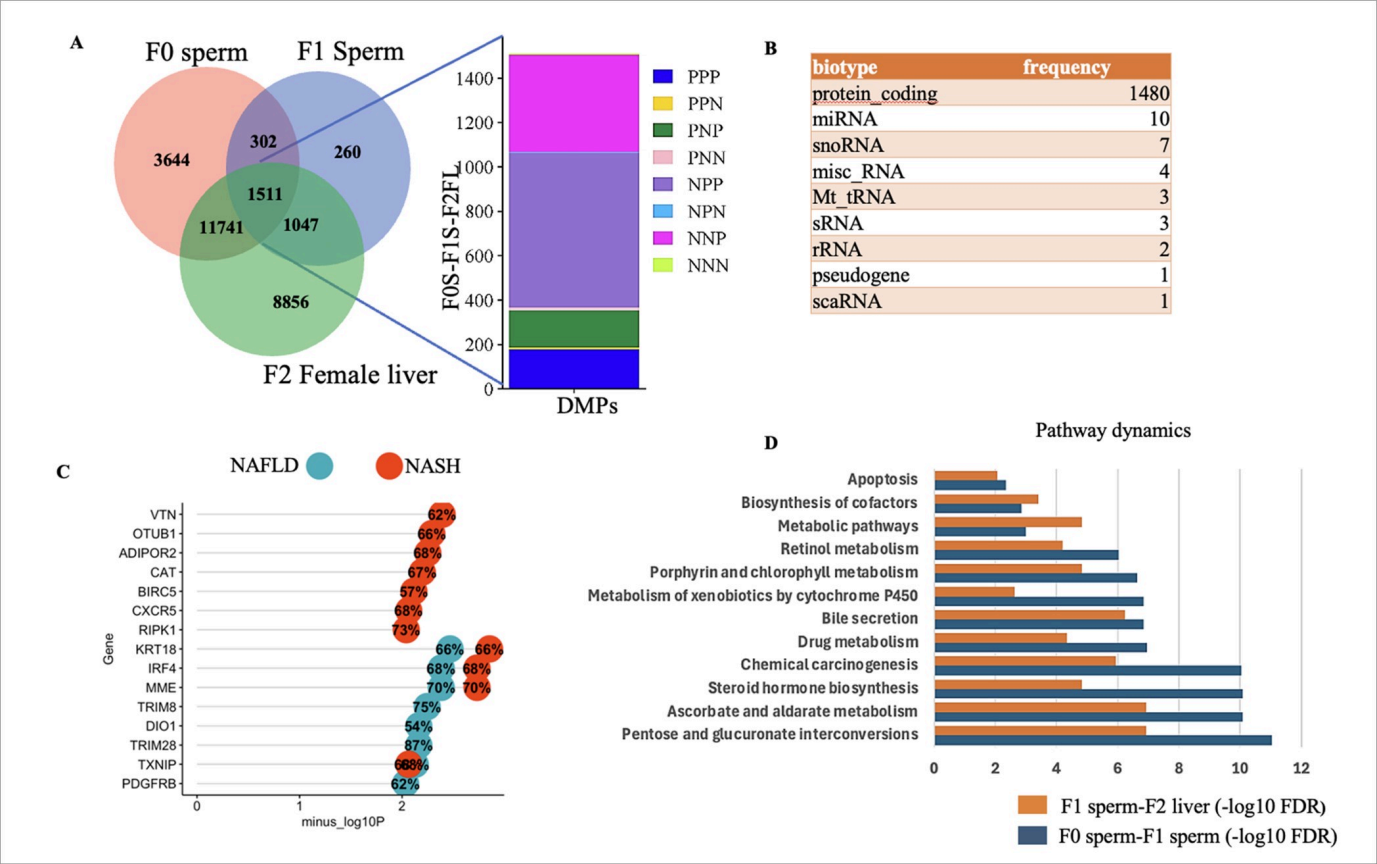
Genes associated with shared DMPs among F0 sperm, F1 sperm, and F2 female livers are linked to NAFLD and NASH. **A**Venn diagram showing the overlap of DMPs among F0 sperm, F1 sperm, and F2 female livers. The common DMPs are further categorized as P (hypermethylated) or N (hypomethylated) in each individual sample. **B** Frequency table showing the gene biotypes linked to shared DMPs across all generations (N=1511). **C** Lollipop plot illustrating the association of shared DMPs with NAFLD and NASH, with the percentage likelihood of disease causation displayed inside the circle. **D** Comparison of pathways associated with common DMP-related genes (DMPGs) between F0 sperm-F1 sperm and F1 sperm-F2 livers of the BPA-lineage fish.

The livers of BPA lineage females developed a more severe disease phenotype with the expression of genes linked to NAFLD, as described earlier. Therefore, the functional role of inherited DMP-associated genes (DMPGs) was thoroughly investigated in F2 livers. The 1511 DMPGs that were persistent across the F0 sperm, F1 sperm, and F2 livers were used for downstream analysis to determine their role in NAFLD-NASH (Fig. 3A). Like those in males, the inherited DMPs in F0 sperm, F1 sperm, and F2 female livers were predominantly classified into PPP, PNP, NPP, and NNP categories (Fig. 3A). In the livers of the F2 females, the inherited DMPGs were relatively evenly distributed across all chromosomes (Supplementary Fig. 2). These DMPGs were associated with various biotypes (Fig. 3B). The majority of these genes were protein-coding genes, although other biotypes, such as miRNAs, snoRNAs, mt-tRNAs, pseudogenes, rRNAs, scaRNAs, and sRNAs, were also identified (Fig. 3B). Among the nonprotein-coding genes, 10 DMPGs were miRNAs, including *ola-mir-142, ola-mir-140, ola-mir-27d, ola-mir-210, and ola-mir-150.* Among these miRNAs, miR-142 and miR-140 have previously been implicated in the pathogenesis of NAFLD[39, 40].

To determine gene–disease associations, VarElect[41] was used with common DMPGs as inputs and NAFLD and NASH as the query phenotypes (Fig. 3C). A stringent criterion of p value < 0.01 and disease association > 50% was applied, considering only DMPGs directly linked to the disease. The analysis revealed that *VTN, OTUB1, ADIPOR2, CAT, BIRC5, CXCR5, and RIPK1* were linked to the NASH phenotype, whereas *TRIM8, DIO1, TRIM28, and PDGFRB* were associated with NAFLD in F2 female livers (Fig. 3C). Additionally, four *DMPGs—KRT18, IRF4, MME, and TXNIP*—were associated with both disease phenotypes (Fig. 3C). To understand the common pathways and how their dynamics change across generations, the common DMPGs between F0 sperm and F1 sperm and between F1 sperm and F2 female liver were selected and individually subjected to pathway analysis (Fig. 3D). The common pathways included metabolic pathways and biosynthesis of cofactors, which were more enriched in F1 sperm-F2 livers than in F0 sperm-F1 sperm. In contrast, pentose glucuronate interconversion, bile secretion, ascorbate aldarate metabolism, and steroid hormone biosynthesis were significantly more enriched in F0 sperm-F1 sperm than in F1 sperm-F2 liver.

### Transcriptional changes in the livers of BPA lineage female offspring were associated with inherited DMPs.

**Figure 4:**
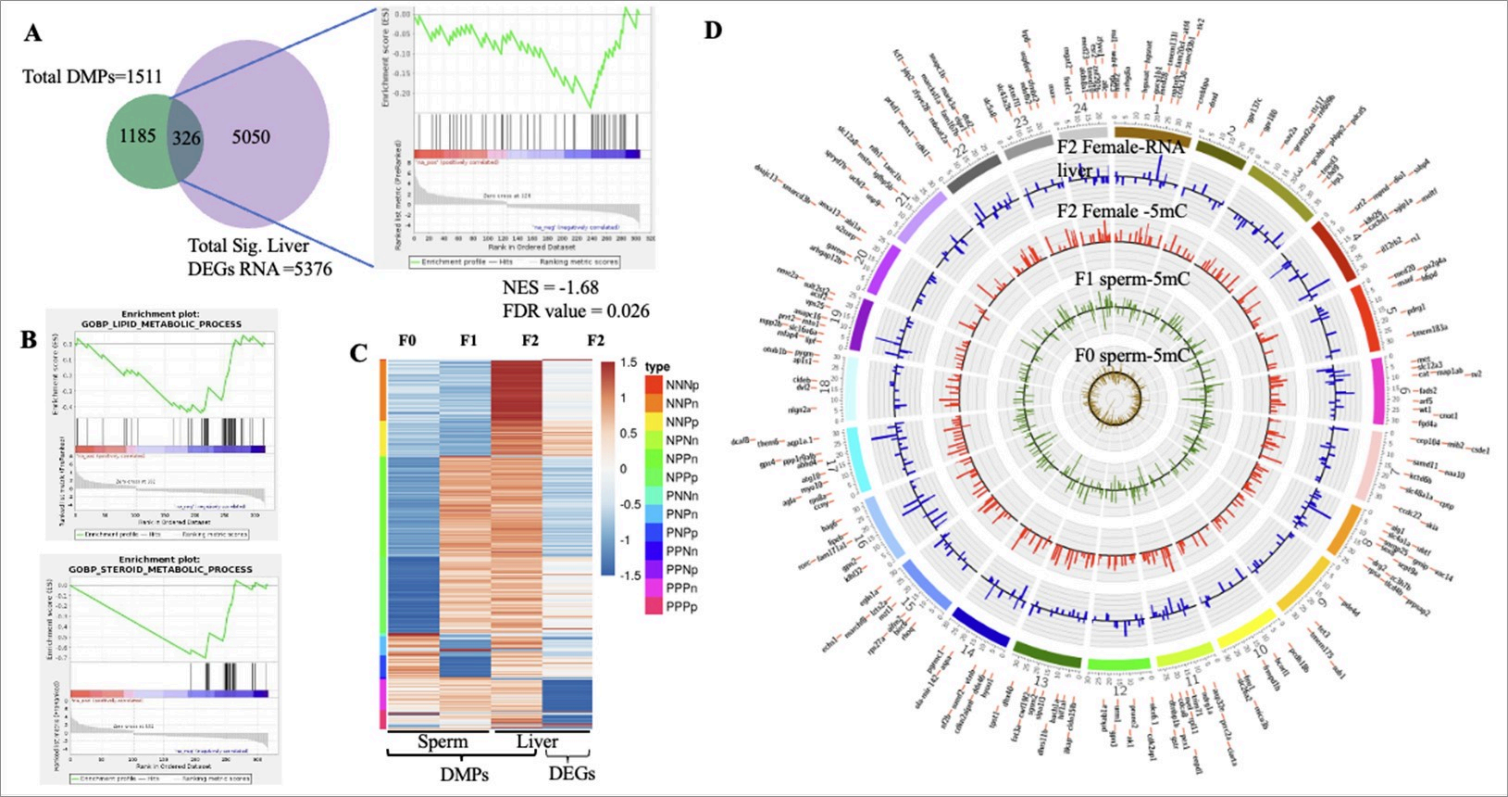
Common inherited DMPs across generations partially overlap with the liver transcriptome, contributing to the development of NAFLD. **A** Venn diagram showing the overlap of common significant DMPs across generations (F0 sperm, F1 sperm, and F2 female liver) and the significant DEGs of the F2 female liver (Total Sig. Liver). On the right side, GSEA of common genes, referred to as differentially methylated and expressed genes (DMEGs; N=326), via the Harmonizome NAFLD dataset is shown. **B** GO enrichment analysis of DMEGs performed via GSEA. **C**Heatmap of DMEGs. The genes are also categorized on the basis of DMP type (hypermethylated = P, hypomethylated = N) and differential expression (p = upregulated, n = downregulated) in the respective samples. **D** Circos plot showing the chromosomal distribution of DMEGs. In the Circos plot, the first three inner circles represent DMPs (F0 sperm, F1 sperm, and F2 somatic cells) and the outer circle contains DEGs in the liver. Bars extending out of the circle represent hypermethylated promoters or upregulated genes and bars in the inner portion of the circle represent hypomethylated promoters and downregulated genes.

Next, we hypothesized that the sperm-mediated transfer of the DNA methylation pattern into somatic cells could play an associated role in altering the transcriptome in the livers of BPA-lineage females. To determine the correlation between the transcriptional changes associated with these inherited DMPGs, we first examined whether the 1511 overlapping DMPGs were linked to changes in the global gene expression profile in the livers of BPA-lineage females (Fig. 4A). A total of 326 genes were shared between the differentially expressed genes (FDRs = 0.05) in the F2 female livers and the shared DMPGs, referred to as differentially methylated and expressed genes (DMEGs). GSEA was performed via the Harmonizome NAFLD gene set[36]. These DMEGs revealed a significant negative correlation (NES = −1.68, FDR q value = 0.026), suggesting a strong association with NAFLD. In addition, their significant negative correlations were found with lipid metabolic processes and steroid metabolic processes (Fig. 4B). A differential promoter methylation pattern in these 326 DMEGs across generations and their associated differential gene expression patterns in the livers of the F2 females are shown in the heatmap (Fig. 4C), demonstrating dynamic methylation patterns during DMP transfer and highlighting the dynamic nature of the intergenerational transfer of DNA methylation patterns across generations. This included hypomethylated promoters in F0 and F1 germ cells becoming hypermethylated in F2 somatic cells, as well as hypermethylated promoters in F0 shifting to hypomethylation in F1 and reverting to hypermethylation in F2. We also plotted the common DMPs and DEGs on a Circos plot, which demonstrated that these genes were broadly distributed across all the medaka chromosomes (Fig. 4D).

**Figure 5:**
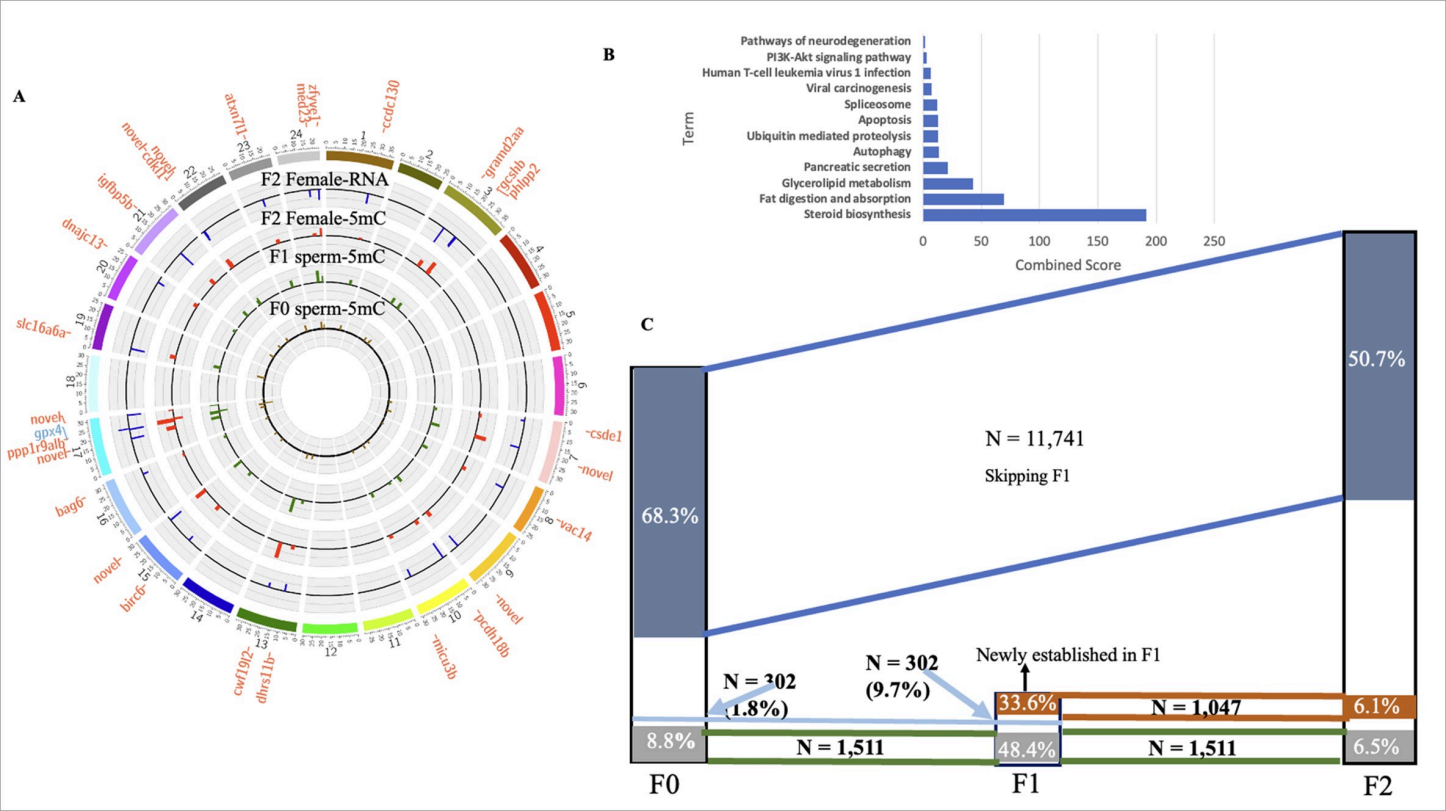
Common canonical genes are linked to NAFLD and the inheritance pattern of DMPs across generations. **A** Circos plot displaying the chromosomal locations of PPPn and NNNp category genes from Fig. 4(C), highlighting canonical genes with consistent hypermethylation (hypomethylation) across all generations and downregulation (upregulation) of gene expression in the F2 liver. PPPn category genes are represented in red, whereas NNNp category genes are shown in blue. **B**Pathway analysis of the PPPn category, as depicted above, reveals pathways associated with NAFLD. **C** A schematic diagram showing the inheritance pattern of DMPs across different generations (F0, F1, and F2), highlighting the DMPs shared between generations and the corresponding percentage in each generation. The numbers next to the generation symbol indicate the total number of DMPs identified to be transmitted.

Our further analysis focused on canonical cases where promoters remained consistently hypermethylated or hypomethylated across F0, F1, and F2, with corresponding downregulated or upregulated gene expression in the F2 liver (Fig. 4C; PPPn and NNNp categories, where n and p indicate downregulated and upregulated DEGs, respectively). These methylation marks might be reprogramming resistant in all three generations. A total of 27 DMEGs with consistent hypermethylation and downregulated gene expression were identified, whereas only one DMEG (*gpx4*) presented consistent hypomethylation with upregulated gene expression (Fig. 5A). To determine the biological role of the 27 shared hypermethylated DMPGs associated with the downregulated DEGs, KEGG pathway analysis was performed on the F2 liver transcriptome database (Fig. 5B). Fat digestion and absorption, glycerolipid metabolism, and steroid biosynthesis were significantly enriched, suggesting their role in the dysregulation of fat digestion (a lipolysis process).

### The dynamic pattern of transmission of DMPs across generations was found

To understand the pattern of DMP transfer across generations, the number and percentages of the shared DMPs were calculated in all generation samples. There were 17198 DMPs in F0 sperm, 3120 in F1 sperm, and 23155 in F2 female livers (Figs. 3A, 5C). A total of 1511 DMPs were common across all three generations, representing 8.8% of F0, 48.4% of F1, and 6.5% of F2 DMPs, indicating consistent inheritance from the F0 to F1 generations and from the F1 to F2 generations. Additionally, 11741 DMPs were shared between F0 and F2, suggesting skipped inheritance, accounting for 68.3% of F0 and 50.7% of F2 DMPs. Furthermore, 1047 new DMPs emerged in the F1 generation and were inherited by F2 generation somatic cells, constituting 33.6% of F0 and 6.1% of F1 DMPs, suggesting that ancestral BPA effects may also manifest in later generations.

## Discussion

Gene–environment interactions result in altered health outcomes that can be heritable. However, the stability of germline transmission in transgenerational inheritance has been in question because these alterations must survive epigenetic reprogramming events in embryos during embryonic development and in germ cells during sex differentiation. Here, we show that ancestral (F0) BPA exposure-induced DNA methylation marks persist in F1 sperm (intergenerational), are transmitted to somatic cells in the liver and are associated with the NAFLD phenotype in the F2 generation. Other chemicals in addition to BPA can also induce sex-specific transgenerational health phenotypes, but the underlying mechanisms are not fully understood[4, 19, 26, 42]. During transgenerational inheritance, skipping phenotype development or the absence of stable transmission of epigenetic alterations across generations are common. We revealed the dynamic nature of sperm-mediated epigenetic inheritance patterns across generations and their association with the transcriptome profile of the liver in the F2 generation, which developed a strong NAFLD phenotype in females. Despite the large number of differential DNA methylation levels and the skipped generation pattern, which has been reported in many transgenerational studies, we found that approximately 1100 unique promoter regions maintained a stable transmission pattern, which can predict future disease (NAFLD) in the liver. The inheritance and NAFLD phenotypes were pronounced in adult females of the BPA lineage. Human studies have also shown that older women are more likely to develop NAFLD than men are[43].

The erasure of DNA methylation is extensive during the reprogramming of primordial germ cells (PGCs), but some DNA methylation marks are believed to be resistant or reestablished after erasure, which may contribute to possible epigenetic inheritance[44]. Our findings revealed that persistent DMP transmission from F0 sperm to the F1 germline and from the F1 germline to the F2 somatic cells of both sexes led to NAFLD, indicating persistent inheritance of gametic DNA methylation during transgenerational inheritance of a phenotypic trait. Ben-Maamar et al.[45] reported the transmission of transgenerational DMR from sperm to morula-stage embryos, showing that most transgenerational sperm DMR sites are retained during the morula stage and are not erased, thus resembling imprinted-like features. This study demonstrated that environmentally induced epimutations in sperm can persist transgenerationally through the morula, a critical stage of early embryonic reprogramming. Epigenetic reprogramming results in global hypomethylation of the genome together with a profound loss of epigenetic memory in primordial germ cells, early embryos, and embryonic stem cells, which underlies naive pluripotency[46]. In medaka, global demethylation continues until the blastocyst stage, and all epigenetic memories in germ cells are supposed to be erased during embryogenesis and PGC reprogramming[47]. In contrast, our findings with inherited DMPs from the F0 to F2 generation challenge the existing dogma of faithful erasure of epigenetic marks during early embryonic and germ cell reprogramming (two waves of reprogramming). The literature suggests that many sites that escape erasure (referred to as “escapees”) are associated with retrotransposable elements, such as intracisternal A particle (IAP) elements in mice and SINE-VNTR-Alu (SVA) elements, as well as subtelomeric and pericentromeric regions[48]. However, by analyzing key genomic regions controlling gene expression, we identified ancestral BPA exposurespecific DNA methylation marks in the promoter region of sperm from the F0 and F1 generations, which persisted in the liver of the F2 generation. This result indicated the presence of genomewide escapees protected against reprogramming-associated demethylation during both the PGC and early embryonic differentiation stages. DMRs in imprinted genes are also resistant to two waves of reprogramming[49]. Since the presence of imprinted genes in medaka has not yet been characterized, we considered those escapees as environmentally imprinted loci in medaka.

We observed a sex-specific transmission pattern of inherited DMPs from germ cells of the grandfather and father (F0 and F1 sperm), who have a history of BPA exposure as individual and primordial germ cells, respectively, to somatic cells of the F2 generation without a history of exposure. Since Mendelian inheritance cannot fully explain the heritable risk for metabolic diseases, environmentally induced epigenetic changes in gametes may represent a risk factor for the development of such conditions in offspring[50]. We identified epigenetically inherited DMPGs associated with NAFLD and NASH phenotypes in both males and females. This suggested that during the reprogramming of germ cells, a differential methylation pattern was introduced in the promoters of NAFLD-NASH genes in F0 germ cells. The genes, mainly *PDGFRB*[51], *TRIM28*[52], *MME*[53], *IRF4*[54], *KRT18*[55], *RIPK1*1[56], *CXCR5*5[57], and *OUTB1*,[58] were consistently inherited from F0 and F1 germlines by the livers of F2 females, suggesting their potential role in promoting the NAFLD-NASH phenotype. These observations indicate that the ancestral BPA-induced aberrant DNA methylation profile in the promoter regions of NAFLD-NASH genes can be transmitted through the germ line, contributing to liver disease in future generations. Consistent with our observations, in high-fat diet (HFD)-fed mouse models, common DMRs were identified in the spermatozoa of F0-HFD-fed mice and their F1 offspring[8, 59]. Previous studies have suggested that not only DNA methylation but also chromatin modifications and noncoding RNAs (such as miRNAs and tRNA-derived fragments) play crucial roles in epigenetic inheritance via germline transmission[60, 61]. Exploring other epigenetic modifications involved in the BPA-induced epigenetic inheritance of NAFLD would simplify the complexity of the epigenetic inheritance of the disease phenotype.

Among the factors regulating gene expression, DNA methylation in promoters has an inverse relationship with gene expression and a positive association with various disease pathways [62, 63]. To understand the contribution of ancestral sperm DNA methylation to disease pathways, we identified overlapping disease-specific pathways in common DMPs from F0 sperm to F1 sperm and F1 sperm to F2 liver. These pathways included pentose and glucuronate interconversion, ascorbate and aldarate metabolism, steroid hormone biosynthesis, cytochrome P450 metabolism, and bile secretion, with greater enrichment observed in F0 sperm to F1 sperm than in F1 sperm to F2 liver. Among these, pentose and glucuronate interconversion[64], cytochrome P450 metabolism[65], and bile secretion[66] are specifically linked to liver disease. Conversely, metabolic pathways that are associated with NAFLD pathogenesis[67] and cofactor biosynthesis were more enriched in F1 sperm and F2 liver than in F0 sperm and F1 sperm.

We identified 326 significant DEGs in the liver of F2 females that overlapped with 1511 shared DMPGs in the F0 sperm, F1 sperm, and F2 liver, indicating the potential link of DNA methylation-dependent transcriptional changes in the liver of F2 females. GSEA of the 326 genes, known as DMEGs, revealed a significant correlation with genes expressed in NAFLD. These genes were also enriched in related GO terms linked to NAFLD pathogenesis, such as lipid metabolic processes, lipid binding, and steroid metabolic processes. Among the 326 DMEGs, 27 epigenetically inherited DMPGs in the livers of F2 females hypermethylated across all generations presented significant downregulation of *birc6*[68], *pcdh18b*[69], *igfbp5b*[70], and genes linked to NAFLD and advanced liver disease. In contrast, the *gpx4* gene, which is involved in the oxidative stress response, consistently maintained persistent hypomethylation in F0 sperm, F1 sperm, and F2 female livers, along with a significant increase in gene expression in the liver. siRNA-induced knockdown of GPX4 resulted in reduced lipid stress, ferroptosis, and cell damage, all of which are linked to the progression of metabolic-associated fatty liver disease[71]. Fat digestion and absorption, glycerolipid metabolism, and steroid biosynthesis were enriched in the canonical list of 27 downregulated genes with hypermethylated promoters that were commonly inherited through F0 sperm and F1 sperm in the BPA-exposed lineage. The literature suggests that dysregulation of fat digestion and absorption[72] and glycerolipid metabolism[73] are linked to NAFLD pathogenesis. We identified three signature genes—HSD17B14, AKR1B10, and RGS4—with fold changes in expression similar to those observed in advanced stages of NAFLD in human patients in the female liver of the BPA lineage medaka[37].

## Conclusions

In summary, we demonstrated in medaka that BPA-induced methylation marks are transmitted across generations, leading to severe NAFLD phenotypes in females that are not directly exposed to BPA, suggesting that epigenetic changes introduced by ancestral BPA exposure can persist in unexposed generations, even after BPA is removed from the environment. A total of 1156 DMPs altered in the F0 generation were transmitted to the F1 generation and then to the F2 generation. The epigenetic memory introduced in F0 was linked to sex-specific NAFLD phenotypes in subsequent generations, suggesting the potential for predicting the phenotypic outcomes of environmental exposures. Overall, the results suggest that sperm exhibit plasticity in reconfiguring DNA methylation in response to environmental chemicals and that BPA-induced epigenetic marks may resist reprogramming, facilitating transgenerational inheritance of NAFLD phenotypes. The present findings provide evidence of the stable vertical flow of environmentally induced germline epigenetic modifications and their horizontal flow in somatic cells, with prediction of pathways associated with nonalcoholic fatty liver disease.

## Methods

### Animal maintenance, ancestral exposure, experimental design, and sampling

This study was conducted by using the Hd-rR strain of medaka[74]. Compared with mice and humans, medaka fish maintain similar epigenetic reprogramming in terms of germ cell reprogramming[75], and they can also serve as alternative animal models to study human nonalcoholic steatohepatitis[76]. These fish have been previously demonstrated to develop transgenerational reproductive and metabolic transgenerational diseases due to environmental exposure[1, 32, 77, 78]. The protocol of the transgenerational exposure study, procedure for handling, and fish and euthanization were approved by the Institutional Animal Care and Use Committee (IACUC) of the University of North Carolina Greensboro (#20 − 002). By using 20 L glass aquaria on a light − dark cycle of 14:10 h with a recirculatory water system with an exchange of 25% water every 4 h at 26 ± 1°C, medaka fish were maintained under laboratory conditions. Otohime granular food (Reed Mariculture) and newly hatched brine shrimp (*Artemia* nauplii) were used as feed. In this study, a BPA concentration of 10 μg/L was selected as the ancestral exposure concentration since this concentration is environmentally relevant to more than 50% of the world’s ecosystems and was found to induce NALFD in medaka[79, 80]. The protocol for generating the dosing solution of BPA was previously described, and the concentration of BPA in the exposure solution was measured by mass spectrometry[81, 82]. A window of exposure spanning eight hours postfertilization (hpf) to fifteen days postfertilization (dpf) was chosen for this study. To avoid embryonic stem cell differentiation, BPA exposure was initiated at 8 hpf and continued for 15 days, which coincides with the critical period of sex determination and liver differentiation in medaka[83, 84]. After BPA exposure was complete, the F0 generation (exposed individuals) and subsequent generations (offspring) were raised in clean water without exposure to BPA. The experimental fish and their subsequent generations’ offspring were never exposed throughout their entire lives. A total of six pairs of F0 (first generation) fish were bred at 120 days of age to generate F1 offspring (second generation, intergenerational). The same breeding approach was used to generate subsequent generations up to F2 (third generation, transgenerational). Each generation of the BPA- and control-lineage fishes was maintained separately. The experimental design included three biological replicate tanks per exposure group, and each biological replicate received embryos from separate breeding pairs. Nine males and nine females from both the BPA lineage and the control lineage were selected for sampling. A buffered MS-222 solution (250 mg/L) was used as an anesthetic. Liver samples obtained from F2 generation fish were used for histology and molecular analysis. DNA and RNA from three fish from each biological replicate were pooled for RNA and DNA methylome library preparation. Similarly, sperm DNA from BPA-treated and control lineages was used for methylome sequencing.

### Genomic DNA and total RNA extraction

Genomic DNA and total RNA from the sperm and liver samples, respectively, were extracted via the All Prep DNA/RNA/miRNA Universal Kit (QIAGEN, Cat No: 80224) following the manufacturer’s guidelines and a previously outlined procedure[85]. The quantification of genomic DNA and total RNA was performed via Nanodrop 2000 and Qubit, and the samples were stored at − 80°C for subsequent analysis.

### Whole-genome bisulfite library preparation and data analysis

The process of preparing whole-genome bisulfite sequencing libraries was outlined in a prior study[85]. In summary, libraries were constructed via the NEBNext^®^ Ultra^™^ II FS DNA Library Prep Kit (NEB, E6177), following the provided user manual. For each sample, 100 ng of genomic DNA was supplemented with 0.5% unmethylated *E. coli* DNA as an internal control.

The “nf-core/methylseq” (v1.6.1) automated bioinformatic pipeline[86] for methylation (bisulfite) sequencing data was used to analyze all the samples. The “nf-core” bioinformatics pipelines are community-curated, highly scalable, and entirely reproducible. In particular, the “nf-core/methylseq” pipeline’s “Bismark workflow” was used. It performs substantial quality control on the outcomes after preprocessing the raw data from Fastq inputs and utilizes Bismark[87] to align the reads on the medaka genome (assembly ASM223467v1). The Bismark coverage files, which describe the methylation percentages and overall coverage at each CpG location, of each individual sample were collected for further downstream analyses via the methylKit R package[88]. The samples of all the generations were processed in the same way. First, a minimum coverage of 3 at CpG sites was required for them to be considered validly covered sites. Next, for genome-wide tiling window analysis utilizing the methylKit, a tiling window size of 100 and a step size of 100 were employed. For promoter analysis, the region ranging from − 3000 bp (upstream) to + 300 bp (downstream) relative to the transcription start site (TSS) was assessed for differential methylation. In the methylation analyses, significance was defined as an FDR < 0.1. In the differentially methylated promoter (DMP) analysis, hypermethylated and hypomethylated DMPs were annotated as “P” and “N”, respectively.

### RNA-seq library preparation and data analysis

The RNA-seq libraries were constructed following the manufacturer’s protocol with the NEBNext Ultra II RNA Kit. Subsequently, sequencing was carried out on an Illumina HiSeq X system (Novogene Corporation, CA, U.S.A.) via a 150 bp paired-end sequencing approach. The initial processing of the reads was conducted via fastp 0.23.2, a comprehensive FASTQ preprocessor that performs multiple tasks, including quality assessment, adapter trimming, and quality filtering in a single pass of the FASTQ data[89]. The processed reads were then aligned to the Medaka genome (ASM223467v1) via STAR v2.7.7a[90]. DESeq2 (v1.34.0) was used for differential expression analysis. In the transcriptome analyses, significance was defined as an FDR < 0.05. Additionally, downstream transcriptome analysis and data visualization were performed via Shiny GO[91], as were enrichment analyses utilizing GO terms (http://www.geneontology.org) and KEGG pathways (http://www.genome.jp/kegg). VarElect[41] was used to determine associations with NAFLD and NASH. The up- and downregulated genes identified via RNA-seq were coined “p” and “n”, respectively, in the results. For the enrichment analysis of NAFLD-related genes, a list of 2,750 genes associated with nonalcoholic fatty liver disease was utilized. This list was obtained from the curated CTD (Comparative Toxicogenomics Database) Gene–Disease Associations dataset and accessed via the Harmonizome (v3.0) database[36] (https://maayanlab.cloud/Harmonizome/gene_set/Nonalcoholic+Fatty+Liver+Disease/CTD+Gene-Disease+Associations). A previously published 25-gene signature associated with advanced NAFLD (including steatohepatitis and fibrosis)[37] in human patients was also utilized to assess the association of liver RNA-seq DEGs with advanced NAFLD. Ensembl BioMart was used to identify orthologous genes between human and medaka species, enabling a comparison of genes across the two species[92].

### Data, Materials, and Software Availability

The raw sequence data generated via whole-genome bisulfite sequencing of DNA and bulk RNA-seq have been submitted to the National Center for Biotechnology Information (NCBI). The methylome and transcriptome of the liver data have been submitted to NCBI, and the accession numbers are GSE285665 and GSE252744, respectively. All other data are included in the manuscript and/or supporting information.

## Data Availability

The raw sequence data generated via whole-genome bisulfite sequencing of DNA and bulk RNA-seq have been submitted to the National Center for Biotechnology Information (NCBI). The methylome and transcriptome of the liver data have been submitted to NCBI, and the accession numbers are GSE285665 and GSE252744, respectively. All other data are included in the manuscript and/or supporting information.
